# Pay for performance: an analysis of the context of implementation in a pilot project in Tanzania

**DOI:** 10.1186/1472-6963-14-392

**Published:** 2014-09-16

**Authors:** Anna Elisabet Olafsdottir, Iddy Mayumana, Irene Mashasi, Ikunda Njau, Masuma Mamdani, Edith Patouillard, Peter Binyaruka, Salim Abdulla, Josephine Borghi

**Affiliations:** Ifakara Health Institute, Plot 463, Kiko Avenue Mikocheni, Dar es Salaam, Tanzania; Department of Global Health and Development, London School of Hygiene and Tropical Medicine, 15-17 Tavistock Place, London, WC1H NSH UK

**Keywords:** Paying for performance, Health system, Contextual factors, Health workers motivation, Low income countries

## Abstract

**Background:**

Pay for performance schemes are increasingly being implemented in low income countries to improve health service coverage and quality. This paper describes the context within which a pay for performance programme was introduced in Tanzania and discusses the potential for pay for performance to address health system constraints to meeting targets.

**Method:**

40 in-depth interviews and four focus group discussions were undertaken with health workers, and regional, district and facility managers. Data was collected on work environment characteristics and staff attitudes towards work in the first phase of the implementation of the pilot. A survey of 75 facilities and 101 health workers were carried out to examine facility resourcing, and health worker employment conditions and job satisfaction.

**Results:**

Five contextual factors which affect the implementation of P4P were identified by health workers: salary and employment benefits; resource availability, including staff, medicines and functioning equipment; supervision; facility access to utilities; and community preferences. The results suggest that it is important to consider contextual issues when implementing pay for performance schemes in low income settings. It highlights the importance of basic infrastructures being in place, a minimum number of staff with appropriate education and skills as well as sufficient resources before implementing pay for performance.

**Conclusion:**

Health professionals working within a pay for performance scheme in Tanzania were concerned about challenges related to shortages of resources, limited supplies and unfavourable community preferences. The P4P scheme may provide the incentive and means to address certain constraints, in so far as they are within the control of providers and managers, however, other constraints will be harder to address.

## Background

The majority of low income countries are far off target to meet the Millennium Development Goals (MDGs) 4 and 5; only 31% are on track to meet the child health MDG4 and 9% the maternal health MDG5 [1]. Investments in strategies to help countries reach these targets through increased coverage of quality health services are increasing. Pay for performance (P4P) has been identified as one of these strategies. P4P schemes provide financial incentives to health care providers that are tied to the achievement of service coverage and/or quality improvements [2]. P4P generally involves intense performance monitoring, improvements to health information systems and greater financial autonomy for providers and increased accountability within the health system [3].

The overall assumption is that P4P will motivate health workers and their managers to increase productivity and quality of care, and ultimately strengthen the health system [3, 4]. Whilst an appropriate incentive package is essential for the success of P4P schemes, it has been argued that the environment in which it is implemented is also critical, ideally featuring clear governance structures promoting participation and planning at all levels, sustained and supportive management and supervision systems as well as the availability of appropriate equipment, essential medicines and other medical supplies [5, 6].

In 2013, over 30 low and middle income countries were implementing P4P schemes, with around half of these in Africa [7]. Yet, evidence on the impact of P4P on access to quality health care in low and middle income countries is still limited; positive effects have been reported in Rwanda and Congo [8, 9] whilst no effects or negative effects were observed in Burundi and Uganda [10, 11]. Evidence of P4P effects on health workers’ motivation and professionalism is also scarce [11]. Furthermore, the methodological quality of studies is often poor [12]. Importantly, there has been limited research into the context in which P4P schemes have been implemented and there is a poor understanding of how the context may affect P4P implementation and overall effectiveness.

Against this background, this paper describes the context within which a P4P programme was introduced in Tanzania and discusses the potential for P4P to address system constraints to meeting targets.

## Methods

### Study setting

**Figure 1:**
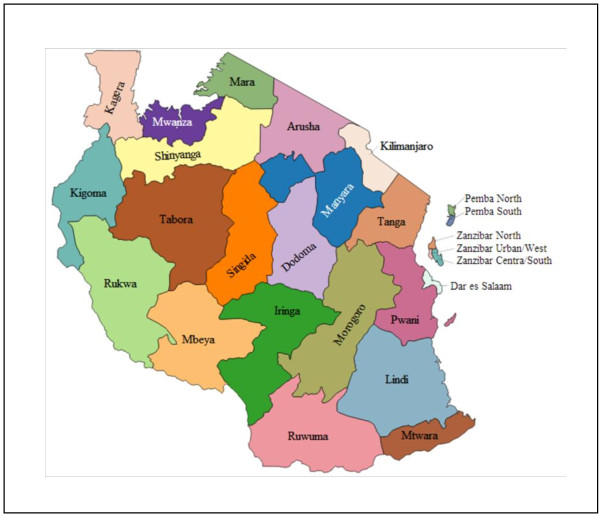
**Regions in Tanzania.** Pwani is in the eastern part of the country. Source: Gregor Aisch on Wikimedia Commons with full permission of usage.

Tanzania is a low income country with gross domestic product (GDP) per capita (PPP) of $1,775 USD, where total health expenditure accounts for 7% of GDP [13]. Officially, P4P was launched in January 2011 in Pwani region of Tanzania (Figure 1). However, the training, that informed health workers about the content of the scheme, was not done until the second half of 2011 and the first payments were made in the first quarter of 2012. A couple of years prior to the start of the scheme, the government launched a national P4P programme, but this was never fully implemented, meaning health workers are less likely to have changed their behavior in response to the scheme until the first payment was made. Therefore, although health workers were aware of the scheme, they only started to respond to the scheme in the second quarter of 2012. The scheme is implemented by the Tanzanian Ministry of Health and Social Welfare, with technical assistance from the Clinton Health Access Initiative and financial support from the Government of Norway. All facilities (public, private and faith-based) providing reproductive and child health (RCH) services in Pwani were eligible to participate in the pilot. A series of maternal and child health services including, for example, institutional deliveries, and provision of two doses of Intermittent Preventive Treatment (IPT) for malaria during antenatal care are incentivised by the scheme [14]. 75%-90% of facility bonuses are paid to health workers with the remainder going to facility strengthening (drug, supply purchase or minor renovation). Bonus payments account for approximately 10% of the average monthly salaries for RCH workers. In addition, district and regional staff are eligible for bonus payments subject to the performance of the facilities in their district and region and timely and complete compilation of reports. Fund pay-outs are administered by the National Health Insurance Fund, and made every six months based on performance in the previous cycle.

### Study design

Process and impact evaluations of the P4P programme were carried out. As part of the process evaluation, data was collected from a sample of districts over three time points during the life time of the pilot [14]. Here we present the findings from the first round of qualitative data collected from December 2011 to March 2012. We report findings from in-depth interviews and focus group discussions with health workers, and regional, district and facility managers on their perceptions of the environment in which P4P was introduced and its influence on implementation. Qualitative findings presented are supplemented by results from facility and health worker surveys carried out in January 2012, as part of the evaluation of the impact of P4P on service use and quality.

Five out of the seven intervention districts were sampled for qualitative data collection, including peri-urban and rural districts and a remote island. Three health facilities participating in the pilot were purposively selected from each of the five districts to ensure representation of each level of care and ownership type (public, faith-based and private), with a total sample of 15 facilities (6 dispensaries, 4 health centers and 5 hospitals; 13 government owned facilities, one faith-based and one private). Interviews were conducted with health workers (n = 27), and with district managers (n = 13). Focus group discussions were held with Health Facility Governing Committee members responsible for facility resource management, from three government facilities and with regional managers.

Qualitative data was collected by four Tanzanian social scientists working in pairs at the time of the first cycle of payment. Interviews were conducted in Kiswahili and tape recorded. Audio tapes were transcribed and translated into English by the researchers who conducted the interviews. Data was entered and analysed in Nvivo 9 using thematic content analysis.

A survey of 75 facilities and 101 health workers were carried out in Pwani region. Of the 75 sampled facilities, the majority were dispensaries (71%), followed by health centres (21%) and hospitals (8%). The majority of facilities (83%) were owned by the government, 12% by faith based institutions and 5% were parastatal/military facilities.

Most of the health workers interviewed (71%) were female and just above half (53%) had college level education or above. The sample of health workers was roughly equally distributed between clinical cadres (44%) and nursing cadres (45%) with the remainder being paramedical cadres.

The survey measured basic service provision within the facility (staffing levels, opening hours, facility management, as well as facility infrastructure, drug and equipment availability) in the 12-month period before P4P was implemented. Availability of equipment was based on provider recall. Availability of drugs and supplies was based on review of the drug register/stock cards at facilities.

Facilities were sampled from those that were eligible to participate in the scheme and included all eligible hospitals, health centres and non-public dispensaries. Public dispensaries were sampled at random with probability proportional to the number of public dispensaries in a given district. The health worker survey measured the P4P effects on providers’ working conditions and attitudes towards work at the selected facilities. Health workers were sampled at random from each facility from those who were on duty at the facility on the day the interviewers were present. Data was collected from facility staff members by 8 teams of 7 interviewers and 1 supervisor. Data was collected using Samsung Galaxy devices (health worker survey) and on paper (facility survey). Data was analysed using Stata 12. Ethical approval was received from the Ifakara Health Institute institutional review board approval number: 1BI1IRB/38 and the ethics committee of the London School of Hygiene & Tropical Medicine. Written informed consent was obtained from all respondents.

## Results

During qualitative interviews, health workers identified five contextual factors related to the implementation of P4P: 1) salary and employment benefits; 2) resource availability, including staff, medicines and functioning equipment; 3) supervision; 4) facility access to utilities, and 5) community preferences or attitudes.

### Salary and employment benefits

Results from the health worker survey reveal that the average monthly salary of health workers was 570,763 ($357 USD) Tanzanian Shillings (sd = 911,496) ($570 USD) and that only 7% of interviewed health workers were satisfied with their current salary. This situation may have been the result of unpaid worked overtime as health workers reported working 74 hours per week (sd = 50) whilst being contracted for an average of 62 hours (sd = 53) (Table 1).

**Table 1:** **Working hours, salary and benefits for health workers**

Variable (n = 101)	Mean	Standard deviation
Number of working hours contracted per week, Mean [SD]	62	53
Number of working hours worked last week, Mean [SD]	74	50
Monthly net salary in Tsh, Mean [SD]	570,763	911,487
Received salary payment with delays, %	36	
Number of days salary payment were delayed last month, Mean [SD]	5	5
Satisfied with salary, %	7	
Satisfaction with employment benefits, %	21	
Satisfaction with living allowances, %	27	
Has other job/activity with income, %	27	

Delays in receiving salaries were commonly mentioned, with more than one third of respondents reporting an average of five days (sd = 5.5) delays for the month preceding the survey. More than one quarter (27%) of respondents also reported having another source of income (Table 1).

The qualitative interviews revealed that overtime payments and travelling allowances were not paid due to lack of resources.

*“Shortage of budget affects us in the sense that … we cannot pay entitlements to our staff, for instance we have not been able to pay on-call allowances and working overtime for about six months which is very de-moralizing”.*

In-charge in a health centre, February 2012

*“These files here [points at files on the table] are claims for extra duties and travelling allowances… Staff haven’t been paid. … if a whole year passes without [staff] being paid they get demoralized”.*

Council manager, January 2012

### Resource availability: staff, medicines and functioning equipment

The facility survey disclosed that the availability of clinical and nursing staff was below the government stipulated norms at most of the primary level facilities visited (Table 2). Less than 20% of primary level facilities had the required number of clinical staff (19% of health centres, 11% of dispensaries); 25% of health centres and 30% of dispensaries had the required number of nurses.

**Table 2:** **Facilities resources in the baseline survey**

Resources (n = 75)	%
*Staffing: percentage of facilities meeting the ministry’s norms:*	
*Clinical staff*	
Hospitals	5
Health centres	3
Dispensaries	6
*Nursing staff:*	
Hospitals	NA
Health centres	4
Dispensaries	16
*Paramedical staff:*	
Hospitals	NA
Health centres	10
Dispensaries	35
*Utilities:*	
Electricity	69
Water	73
Toilet	97
*Stock Outs (% facilities reporting in past 90 days)*	
Intermittent Preventive Treatment (IPT)	27
Anti-retroviral drugs (ARV)	70
*Functioning Equipment*	
Facilities reporting broken equipment	29
Facilities reporting broken equipments disrupted service delivery	12

Note: NA = data not available.

The qualitative interviews indicated that shortage of clinical and nursing cadres in primary level facilities may have led to paramedical and other staff sometimes stepping in to deliver services, despite a lack of training to do so.

*“Sometimes you find yourself being alone providing services in the facility. Therefore it is very difficult to perform the work perfectly the way we learned. Sometimes you are forced to be a “doctor”, which you did not get training for … but it [the shortage of professional staff] forces you to do that”.*

Health worker in a dispensary, January 2012

Drug stock outs were common at facilities: over a quarter of facilities surveyed (27%) reported stock outs of intermittent preventive treatment (IPT) in the 90 days preceding the survey and 70% reported stock outs of anti-retroviral drugs (ARVs) (Table 2). Only one third of health workers surveyed said they were satisfied with the availability of medicines, and less than one quarter (22%) with medical supplies (Table 3).

**Table 3:** **Health workers satisfaction with their work and working conditions**

Variable (n = 100)	%
Satisfied with medicines availability	33
Satisfied with medical supplies availability	22
Satisfied with functioning equipment availability	17
Satisfied with physical condition of the facility	38
Satisfied with own ability to provide high quality care given current working condition	43
Glad to work in the facility	51
Job makes me feel good about myself	85

Concerns about drug shortages were voiced during qualitative interviews:

*“Another challenge is the shortage of drugs. This [the shortage of drugs] makes us fail, during the exercise of pay for performance. Many drugs are not available in the health facilities… SP [Sulfadoxine/pyrimethamine] malaria pills were not available and they are important in meeting the pay for performance targets".*

District manager, January 2012

One reason for drug shortages mentioned by several interviewees was a weakness in the operation of the domestic drug distribution chain, resulting in insufficient quantities of drugs being delivered, and sometimes a mismatch between what was ordered and what was received.

*“The problem is with the MSD [Medical Store Department]. You may receive a drug kit, but within the drug kit you find some items are missing. For example, you may have ordered 10 items but you only receive 8 items, the rest is missing, yet the money has been paid and will not be refunded. You will have to wait for the missing drugs to come with the next order, but you can’t be sure ….”*

District manager, January 2012

*“You’ll find that the doctor orders the drugs we want but the problem is with the person who packs them - this person does not necessarily pack the drugs that we require. For instance, now we have rains and we expect to get a lot of diarrhoea and malaria cases so the doctor orders these drugs but …they may not be supplied. The people who pack the drugs should pack according to the doctor’s request and not their own wish to see their stock to be finished. [It is like] you ordered shoes and the supplier sends you socks - would you sign and say; that ok, I am taking these socks?”*

Health Facility Governing Committee member, December 2011

Lack of equipment or report of broken equipment was commonly noted. Forty percent of facilities reported broken equipment and 52% indicated that this disrupted the provision of services. Only 17% of health workers interviewed reported being satisfied with the availability of functioning equipment (Table 3). During qualitative interviews, the poor quality of furniture in the labour ward was also mentioned:

*“… the mattresses that are used at the labour-ward are also used in the general ward. You know the covers used on those mattresses have the tendency to allow water to penetrate and so after delivery even if the blood will be wiped some will penetrate. The mattresses usually stink and produce a very foul smell”.*

Health Facility Governing Committee member, December 2011

District level managers were concerned about the impact of this situation on staff motivation and retention.

*“Frankly speaking, the performance is not good. This is due to the infrastructure and working equipment problems. Therefore, people have no morale of work. You are used to seeing various instruments when you are at the university, but you find nothing when you come to work. It de-motivates people in performing their duties – we lack working equipment, and if it is available it is outdated”.*

District manager, December 2011

*“You allocate new staff to the facilities but after one year they move away. The doctors come here and soon find out that even though the equipment is here, it is of low quality … so they leave”.*

District manager, December 2011

Just above half of the health workers interviewed said they were glad to work in their facility rather than another facility in the country and 43% reported being satisfied with their own ability to provide high quality of care given the current working conditions. However, 85% said the job made them feel good about themselves (Table 3).

During qualitative interviews, some respondents advised that prior to implementing P4P it would be preferable to ensure the availability of necessary equipment to enable health workers to complete basic tasks.

*“…they [decision makers] could bring equipment and then start to implement the program. From there you can start to assess the person. But you haven’t given the person the equipment and still you want to evaluate him/her. If you haven’t given him/her anything he/she will tell you that I can’t do this because I don’t have equipment…”*

Health worker in a hospital, January 2012

### Supervision

About two thirds (62%) of health workers interviewed during the survey said they had an internal supervision, with 78% reporting their internal supervisor to be the facility in-charge (Table 4). Most (85%) health workers said that the last internal supervision took place within the last month. Nearly all (96%) health workers reported having an external supervisor, most commonly a member of the Council Health Management Team. External supervision visits were reported to be less frequent than internal ones (Table 4).

**Table 4:** **Supervision at health facilities**

Variable (n = 101)	%
Staff with internal supervisor	62
Internal supervisor is the in-charge	78
Received last internal supervision in past 30 days	85
Staff with external supervisor	96
External supervisor is Council Heath Management Team member	81
Received last external supervision in past 30 days	52
External supervisor - check records	39
External supervisor - consultation	3

According to the qualitative data one of the obstacles for providing more frequent supervision visits was a lack of financial resources:

*We do supervision every quarter. Our plan is to do supervision monthly but due to limited budget we haven’t yet done this.*

Council health manager and P4P focal person, January 2012

This was confirmed by a facility in-charge:

*Now they [external supervisors] have reduced the frequency of supportive supervision because they do not have fuel to run the cars. They complain that they have been requesting fuel from the DED [District Executive Director] without any success.*

Facility in-charge in a health centre, December 2011

Furthermore, some of the interviewees perceived the quality of external supervision to be poor, as one health centre in-charge phrased it:

*They [external supervisors] do come for supervision, but to me supervision is about checking how the job goes and to make corrections … but that kind of supervision I haven’t seen … they only come to collect reports. …I haven’t seen them giving us any feedback on how we are doing…*

In-charge in a health centre, January 2012

This situation was also reported by health workers during the survey with 39% of external supervisors reported to check records whilst only 3% gave consultations (Table 4).

### Facility infrastructure and utilities

Of all health workers surveyed, less than 40% reported being satisfied with the physical condition of the facility (Table 3). The facility survey revealed that over 30% of facilities had no electricity (Table 2). During qualitative interviews, facilities without access to electricity were said to rely on gas supplied by the district, notably to refrigerate vaccines. However, the health facility survey revealed that nearly half of facilities reported stock outs of gas for cold storage of vaccines (data not shown). This was believed to be mainly due to limited funding from the central government and transport problems at the district level.

*“… the health facility is using gas containers and to fill those containers depends on funds from the district (Council Health Management Team), whereby the district depends on funding from the government. Now it’s a long time since we last got those funds … and it’s a long time now since they [named facility] have given out any immunizations”.*

District manager, January 2012

Access to clean water was also limited; 27% of facilities did not have access to running water according to the surveyed facilities whilst nearly all (97%) had toilet facilities, however, only 35% reported having a flush toilet. Limited waste disposal facilities and lack of water and electricity was also highlighted during the in-depth interviews.

*“we also have no place to dump the placenta. We normally dump it into the toilet. We have a problem with water … it is too far from here to the water hole, we have to fetch water from a well. … Let them [the government] think about our facility needs, we don’t have water and we need energy here, they might connect the solar …. we use hand-lamps to serve patients during night hours”.*

Health worker in a dispensary, January 2012

### Community preferences

A further issue highlighted by health workers during qualitative interviews was community preferences or attitudes towards the formal health care system. This was reported to apply more to areas where educational levels were thought to be low and perceived to lead to a limited understanding of pregnant women needs for professional assistance. This situation, which is beyond the control of health workers, was believed to potentially affect the ability to attain the P4P targets:

*“… it depends on where you are working … if you are serving people who have education it is very simple. But here the level of education … is low. We are trying to give them [health care users] health education but things remain the same. For example, the issue of pregnant mothers to deliver at the hospital is becoming a problem. Many pregnant women do attend a clinic but when it comes to the stage of delivering they don’t come to deliver at the facility - they deliver at home. This is due to local superstition … we try to educate them, but still nothing has changed. One of the P4P indicators is that pregnant women have to deliver in a health facility – but this is difficult to achieve. It is also difficult to achieve the PV0 [first polio vaccine] vaccination, as one of the P4P indicators - the PV0 is supposed to be offered immediately after the baby is delivered but we cannot do that when the baby is delivered at home”.*

Health worker in a health centre, December 2012

## Discussion

This study identified five categories of contextual characteristics affecting the implementation of P4P, including salary and employment benefits, resource availability, supervision, facility access to utilities, and community attitudes. Firstly, the work load on staff is considerably higher than they are contracted for, which is not reflected in the remuneration; payments are delayed and overtime and eligible allowances are not always paid causing demotivation among staff. Secondly, the majority of primary level facilities do not have the required number of clinical staff. The reasons for staff shortages are multiple including unequal distribution between urban and rural areas [15]; the ongoing effects of a recruitment freeze in public sector workers in the 1990s, and limited capacity of training schools [16]. One of the consequences of insufficient staff members is that staff can be forced to take on work they are not trained for. Thirdly, external supervision providing consultancy is limited and feedback given to health workers is scarce. Fourthly, basic utilities are commonly missing such as electricity and running water. Fifthly, the community preferences are sometimes unfavourable for health workers to attain the P4P goals. The study therefore suggests that it is important to consider contextual issues when implementing P4P schemes in low income settings. It highlights the importance of basic infrastructure being in place, a minimum number of staff with appropriate education and skills as well as sufficient resources.

Health worker shortages are a commonly acknowledged problem in Africa but P4P can be expected to improve the supply of health workers. A study in Belize found an increase in nurse density resulting from a national health insurance scheme with inbuilt performance contracts [17]. Health workers low satisfaction with salaries and employment benefits has been reported in other studies undertaken in Tanzania [18, 19]. This has, to some extent, been seen as being responsible for human resource shortages in rural areas especially for clinical and nursing cadre at primary care levels [15, 20]. P4P has the potential to improve health workers’ motivation, by increasing financial benefits. In the case of Tanzania the bonus payments have accounted for around 10% of health workers average monthly salaries. Higher potential earnings may make a rural posting more desirable for health workers, if the additional earnings will offset the loss in earnings from employment in an urban setting and the more constrained working conditions [21].

Overtime levels were high among surveyed health workers, which is consistent with other studies in Tanzania [22], yet payment for overtime could not be relied upon. By providing facilities with additional funding it is conceivable they may be better able to pay overtime and other allowances. However, P4P also has the potential to increase hours worked if utilisation levels increase and workers have limited spare capacity. Time spent on reporting on performance indicators could represent a significant additional time burden.

High rates of drug stock outs and lack of functioning equipments were identified at baseline consistent with findings from facilities elsewhere in Tanzania [23, 24]. Similar issues have also been reported elsewhere [25, 26]. This affects the possibility for health workers to reach P4P targets as some of the targets are related to providing drugs or vaccines that are out of stock. This further leads to health workers’ dissatisfaction, something which has also been reported elsewhere [27]. Although P4P implementation in Rwanda was in many ways successful, weak facility infrastructure was also found to constrain P4P implementation there [26].

The shortage of drugs is mainly a result of limitations in the central procurement system through the Medical Stores Department (MSD), which supplies drugs to government and non-public facilities. Specifically delays in delivering drugs, a mis-match between drugs ordered and those delivered and stock outs of drugs at the central MSD warehouse have been noted [28]. Although the P4P scheme would not be able to affect the central procurement system, the P4P funds could theoretically alleviate the drug shortage problem if they were used to purchase drugs and supplies from private suppliers. However, facilities require permission from the district council to do so. Similarly certain aspects of facility infrastructure might be improved using P4P resources, such as purchasing new mattresses, or undertaking minor renovation work. Lack of availability of basic utilities such as electricity and running water may be less easy to overcome.

One of the major concerns in relation to P4P is sustainability but in this case the scheme is funded by the Norwegian government. However, the implementation of P4P might stimulate the generation of resources to increase sustainable funds available to health care services. If health care services can be improved with a P4P scheme it could help encouraging community enrolment in the Community Health Fund (CHF), which is a voluntary health insurance scheme for people employed in the informal sector that do not pay tax and have no insurance coverage from their employer. Health workers may also be more motivated to enrol patients in the CHF as part of the CHF revenue goes to the facilities serving the CHF members and can be used to purchase essential drugs and other supplies in order to perform better. It is also possible that facilities increase user charges which would generate additional revenue but could also serve to reduce access among the poorest. A study in Congo reported a 25% increase in user fees following the introduction of a P4P scheme [29].

This research raises the question as to whether those facilities with better baseline resourcing and conditions are better able to meet targets [30]. This question will be addressed during forthcoming outputs of this research.

P4P is based on the assumption that health workers respond rationally to incentives, notably monetary rewards combined with additional funds allocated to facilities; more intense supervision and improved information system. Hence, P4P is assumed to increase health workers motivation to reach targets (extrinsic motivation). However, other factors, which can be related to intrinsic motivation also play a role, such as stimulating health workers confidence, give them feedback and allow them to participate in policy making and planning [5, 31, 32]. Furthermore, it is recognized that P4P-like interventions, with contingent rewards, also have the potential to negatively influence health workers’ intrinsic motivation - if, for instance, health workers perceive they cannot reach targets because of factors outside their control or if they perceive that the reward system is unfair [33, 34]. Continued monitoring of the Tanzanian P4P pilot will improve our understanding of the complexities of health worker motivation and by which process and pathways P4P affects motivation.

## Conclusion

Health professionals working within a pay for performance scheme in a low income setting are concerned about challenges related to systemic constraints, shortages of resources, weak infrastructure and unfavourable community preferences and attitudes. They are concerned that these weaknesses can affect their performance negatively and that they might possibility be an obstacle for them to attain their targets. The implementation of the P4P scheme in Pwani region in Tanzania may provide the incentive and means to address certain constraints, in so far as they are within the control of providers and managers, however, other constraints will be harder to address.
